# Facilitators and barriers to the adoption of mHealth apps for COVID-19 contact tracing: a systematic review of the literature

**DOI:** 10.3389/fpubh.2023.1222600

**Published:** 2023-12-07

**Authors:** Sujarwoto Sujarwoto, Asri Maharani

**Affiliations:** ^1^Portsmouth Brawijaya Center for Global Health, Population and Policy and Department of Public Administration, Universitas Brawijaya, Malang, Indonesia; ^2^Division of Nursing, Midwifery and Social Work, University of Manchester, Manchester, United Kingdom

**Keywords:** facilitators, barriers, mHealth apps adoption, COVID-19 contact tracing, systematic review

## Abstract

**Background:**

Despite the enormous potential of mobile health (mHealth) apps for COVID-19 contact tracing, the adoption rate in most countries remains low. Thus, the objective of the current study is to identify facilitators and barriers of mHealth apps adoption for COVID-19 contact tracing based on existing studies.

**Methods:**

We conducted a systematic review of mHealth studies before December 2021 that evaluate facilitators and barriers associated with the adoption of mHealth apps for COVID-19 contact tracing. We assessed the risk of bias for all included studies using the Cochrane tool. We based our narrative synthesis on the facilitators-barriers to the adoption of mHealth framework comprising seven key factors.

**Results:**

A total of 27 articles were reviewed from 16 countries representing high income countries (France, German, Italy, United Kingdom, United States, Australia, Singapore, Belgium, Republic Ireland, Netherland, Poland, and Japan), middle-income countries (Fiji), and low-middle income countries (India). We identified the main facilitators of mHealth adoption: perceived risks to COVID-19, trust, perceived benefit, social norm, and technology readiness. The main barriers of mHealth adoption were data privacy/security concerns. Among sociodemographic factors, females, lower education, lower-income, and older individual are barriers to adoption in low-middle income countries, while most of those factors were not significantly associated with adoption in a high-income country.

**Conclusion:**

The findings imply that resolving data privacy/security issues while developing trust, perceived benefits, social norms, and technology preparedness could be effective strategies for increasing adoption intentions and app use among the general public. In low-middle-income countries, addressing digital divide is critical to the app’s adoption.

Systematic review registrationhttps://www.crd.york.ac.uk/prospero/display_record.php?RecordID=249500, identifier RD42021249500 (PROSPERO).

## 1 Introduction

Smartphone-based digital (mHealth) contact tracing apps have enormous potential to mitigate the current coronavirus disease (COVID-19) pandemic, given the fact that there are more than 3.5 billion smartphone users worldwide (1). Through mHealth contact tracing apps, we can monitor and track infection chains, provide rapid support and information in the event of an illness or contact with an infected individual, and assist people in quarantine by monitoring their health and adapting information to preventive action. The potential of mHealth contact tracing apps for controlling virus spread led to the development of 47 contact tracing apps in 25 countries (2).

Despite the enormous potential of mHealth for COVID-19 contact tracing, the adaptation rate in most countries remains low. In a best-case scenario, 90–95% of the population must use a contact tracing app to stop the spread of COVID-19 and allow normalcy without physical distancing (3). Contact tracing applications were available on different dates for the countries, and it may affect the adoption rates between countries. Only 9.3% of users in the 13 most populated countries having government-endorsed applications have installed the apps (4). Australia had the greatest adoption rate at 21.6%, followed by Turkey at 17.3%, Germany at 14.4%, India at 12.5%, Italy at 7.2%, Peru at 6.8%, and Japan at 5%. The rest of the countries have a lower than 5% adoption rate (4). This evidence shows a need to understand the facilitators and barriers to mHealth contact tracing apps adoption for effectively controlling the epidemic.

In this context, the objective of our study was to identify facilitators and barriers to the adoption of mHealth apps for COVID-19 contact tracing based on existing empirical studies. We believe that the adoption of contact tracing apps, whether successful or unsuccessful, will have ramifications beyond the current pandemic.

The current pandemic caused by COVID-19 presents the greatest global public health crisis since the 1918 pandemic influenza outbreak (5). The dynamics of infectious diseases, as well as the changing global landscape, point to the possibility of future pandemics following COVID-19 (5, 6). Ongoing research, surveillance, and international collaboration are crucial components of global efforts to anticipate, prevent, and respond to emerging infectious threats. Prior studies showed that technologies, including contact tracing apps, are useful in limiting the spread of COVID-19 (7). Learning from the experience of implementing contact tracing apps during COVID-19 will be potentially useful in mitigating future pandemics. Recent review highlighted the barriers to implementing contact tracing apps during the COVID-19 pandemic, which are potentially useful in the future, including user privacy concerns, transparency and ethical considerations (8). The adoption of contact tracing apps during COVID-19 will serve as a model for future opportunities and challenges in incorporating other digital solutions into clinical practice while maintaining user data privacy. Thus, we hope that our findings will add to the discussion about the use of digital technology to solve health problems, even beyond challenges related to the COVID-19 pandemic. Moreover, the continuous monitoring of contacts and potential exposures using contact tracing apps will provide a surveillance mechanism for public health agencies (9). This data can inform decision-making, resource allocation, and the development of targeted public health interventions.

## 2 Materials and methods

### 2.1 Search strategy

PubMed, Embase, Emcare, Global Health, the Cochrane Library, PsycInfo, and CINHL databases were searched for studies published before December 2021 reporting the implementation of mHealth app for COVID-19 contact tracing. WHO Global Research on COVID-19 database was searched for gray literature.

An example of the search terms used is shown in Box 1, and the full search strategy is available in Supplementary material S1 files. The review was designed in accordance with the Preferred Reporting Items for Systematic Review and Meta-Analysis guidelines and was prospectively registered with PROSPERO (CRD42021249500). PRISMA checklists are available in Supplementary Table S1.

**BOX 1**

Example search terms for the systematic review of facilitators and barriers of mHealth adoption for COVID-19 contact tracing apps.

(Mobile health technology/OR mHealth/OR mobile app*/OR smartphone/OR contact tracing app*)

AND

(COVID*/OR Coronavirus/OR Corona/OR SARS-CoV-2)

AND

(adoption/OR acceptance/OR barrier/OR facilitator/OR intention/OR willingness).

### 2.2 Eligibility criteria

We used the definition of mHealth from the Global Observatory for eHealth (GOe) which defined mHealth or mobile health as medical and public health practice supported by mobile devices, such as mobile phones, tablets, personal digital assistants (PDAs), and other wireless devices (10). mHealth involves the use and capitalization of a mobile phone’s core utility of voice and short messaging service (SMS), as well as more complex functionalities and applications such as general packet radio service (GPRS), third and fourth generation mobile phone telecommunications (3G and 4G system), global positioning system (GPS), and Bluetooth technology (10).

Eligibility was limited to studies where one of the primary objectives was to identify facilitators and barriers to adopting mHealth for COVID-19 contact tracing apps. Eligibility study designs included quantitative studies. We excluded qualitative studies. We only used research articles written in the English language.

### 2.3 Screening, article selection, data extraction and quality assessment

As a first step in the data handling process, titles and abstracts of all papers retrieved by the search strategy were screened for relevance, and those clearly irrelevant were discarded. The full text was downloaded if the result was not clearly relevant. As a second step, two authors (SS, AM) independently assess the eligibility of the studies by using the predefined inclusion and exclusion criteria. Any disagreements on whether or not to include a specific study were resolved by discussion between the two authors. The two authors (SS, AM) then independently extracted data from included articles into an Excel template containing the following headings including (1): study design (2); country (3); the nature of mHealth app evaluated (4); facilitators and barriers; and (5) outcomes measures; and (6) the results for each outcome. The reference list of all included articles was hand-searched for additional relevant articles.

The Cochrane Collaboration’s risk of bias tool was used to assess the risk of bias in all studies (RoB 2). Using the Critical Appraisal Skills Program (CASP) quality checklist appropriate to each study design, two authors (SS, AM) independently assess the possibility of bias in the included studies’ design, conduct and analysis. Any discrepancies will be discussed until a consensus is reached. Although no studies were excluded from the review based on quality alone, studies rated as possessing higher methodological strength were synthesized first. Following this, studies appraised as evidencing methodological limitations were then added to the synthesis to investigate whether these lower-quality studies affect the review’s findings. The CASP tools were selected to identify the quality issues across a wide range of study designs.

### 2.4 Synthesis of results

**Figure 1:**
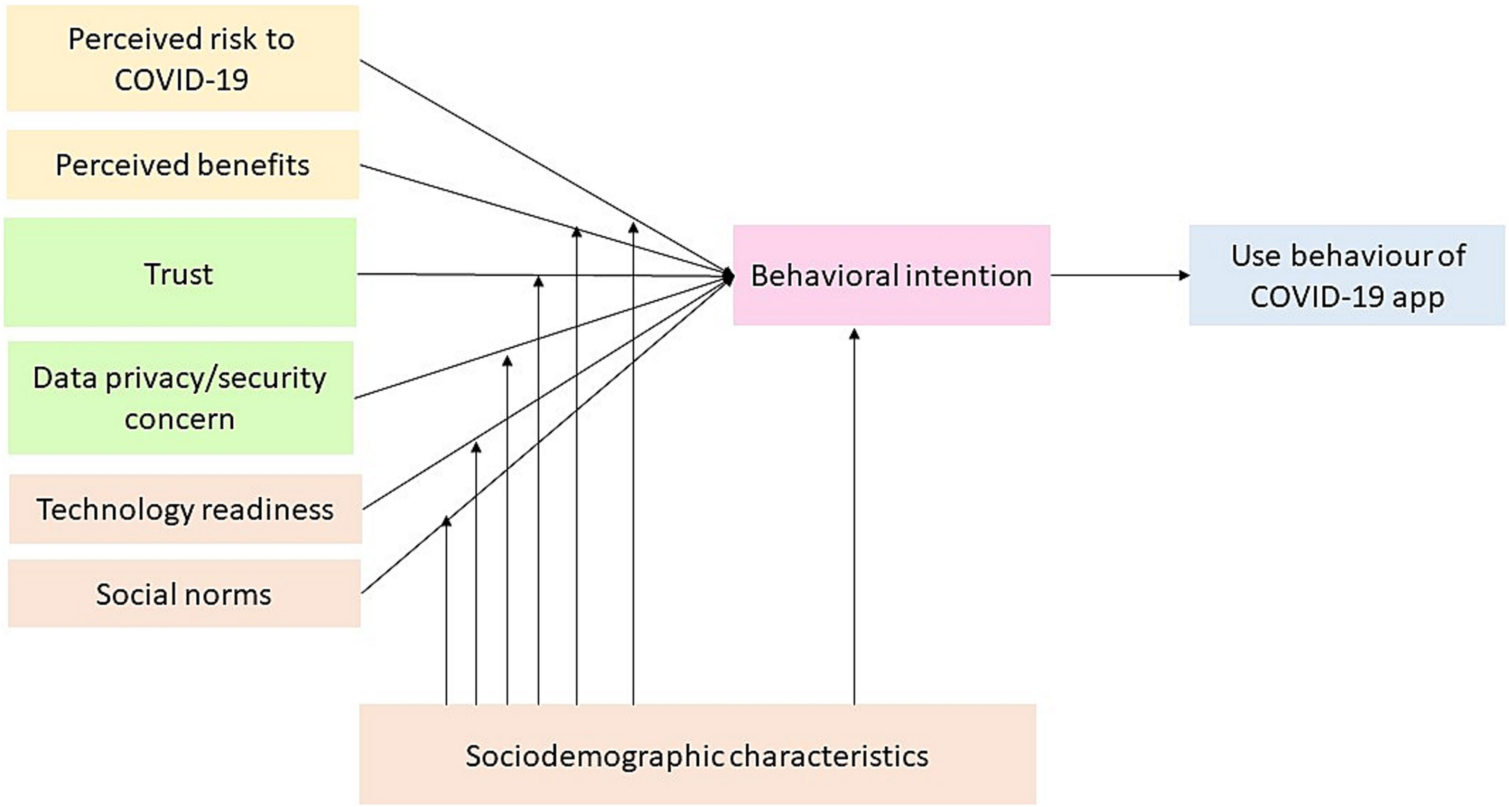
The facilitators and barriers of mHealth adoption framework.

The facilitators-barriers informed a narrative analysis of mHealth adoption framework synthesis from the unified theory of acceptance and use of technology (11, 12), the privacy calculus framework (13), and uncertainty reduction theory (14) (Figure 1). The framework identified seven key factors that explain mHealth adoption (1): perceived risks to COVID-19 (2); data privacy/security concern (3); trust (4); perceived benefits (5); social norm (6); technology readiness; and (7) social-demographic characteristics.

Based on the privacy calculus model, perceived risk is negatively associated with intention and adoption of using mHealth technology, with perceived benefits positively associated with intention and adoption of using mHealth technology (13). According to uncertainty reduction theory, increasing data privacy/security risks is negatively associated with intention and adoption using mHealth technology, with trust positively associated with intention and adoption using mHealth technology (14). The unified theory of acceptance and use of technology suggests that technology readiness is positively associated with intention and adoption of mHealth health technology. This theory also posits that social norms and socio-demographic characteristics can affect mHealth uptake. Socio-demographic characteristics such as age, gender, income, education, ethnicities, living areas, and marital status determine perceived risks to COVID-19, data privacy/security concern, trust, perceived benefits, social norm, technology readiness.

## 3 Results

### 3.1 Study selection

**Figure 2:**
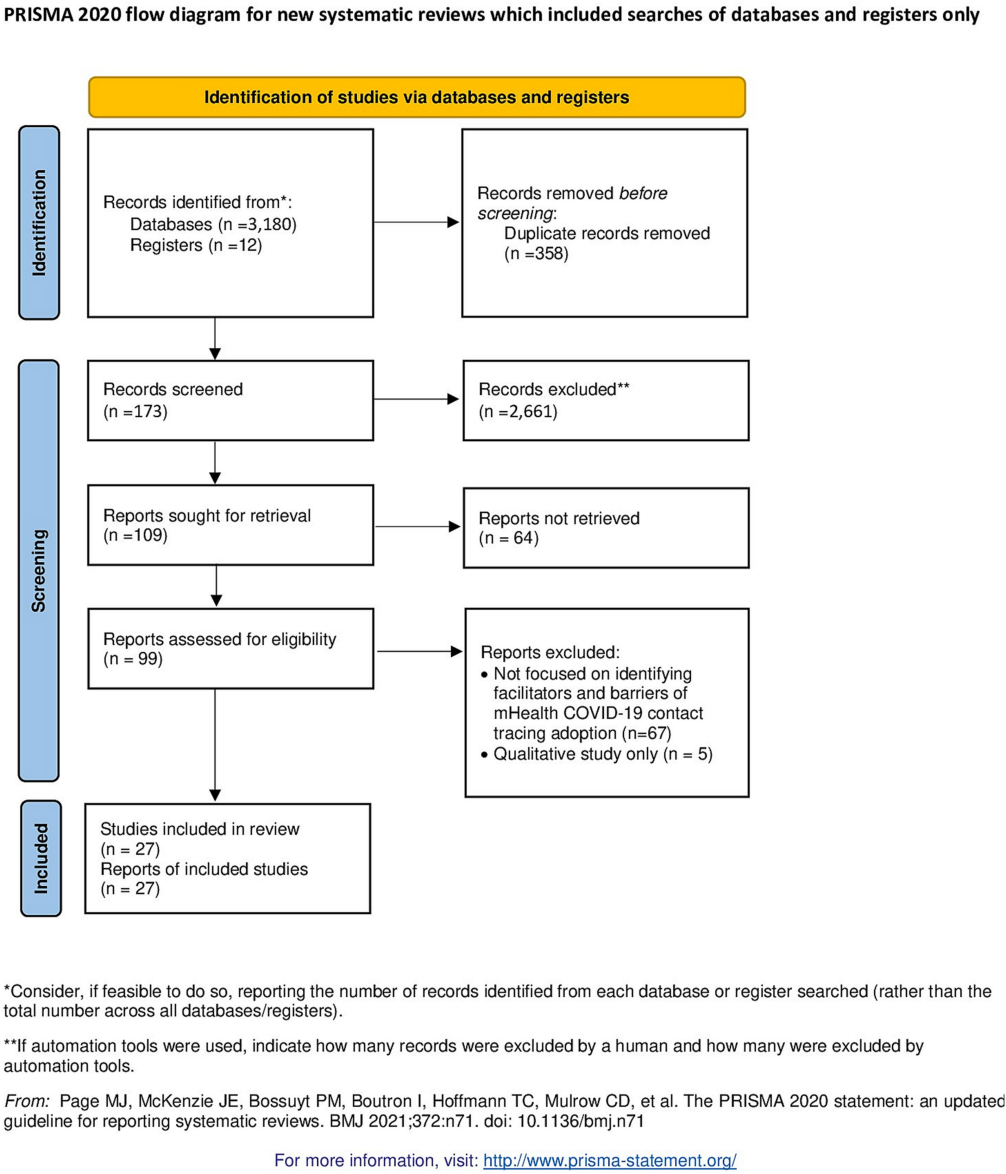
PRISMA flow diagram of a systematic review on facilitators and barriers to the adoption of mHealth apps for COVID-19 contact tracing.

We found 2,834 entries in our initial search, 99 of which matched our inclusion criteria and were subjected to full-text inspection. As a result, 27 studies were included in the final review from 16 countries representing high income economies (France, German, Italy, United Kingdom, United States, Australia, Singapore, Belgium, Republic Ireland, Netherland, Japan, and Polland), upper middle-income economies (Fiji) and lower-middle income economies (India) (Figure 2).

### 3.2 Study characteristics

Detailed characteristics of all included studies are provided in Table 1. Among 27 selected studies, 23 used cross-sectional data either single or repeated and four studies used an experimental design, longitudinal design, conjoint experiment, and discrete choice experiment. Within the cross-sectional design, two studies used cross-country data analyzes, 20 applied single-country analyzes, and one country used repeated cross-sectional data analysis within a city. The number of samples ranged between 288 and 28,246 respondents. Most of the studies use nonprobability sampling methods, such as convenience sampling method, quota sampling method, and purposive sampling method. We identified 10 studies employed certain theories or frameworks including Theory Acceptance Model (TAM), Unified Theory of Acceptance and Use of Technology (UTAUT1 and UTAUT2), Uncertainty Reduction Theory (URT), Health Behavior Models (HBM), Theory Planned Behavior (TPB), Theory of Reasoned Action (TRA), Perceived Behavioral Control (PHC), Hofstede’s Cultural Theory (HCT), Protection Motivation Theory (PMT), and Privacy Calculus Theory (PCT) to develop their hypothesis. There are two final outcomes identified (i.e., app adoption intention and app adoption). Of the 27 studies included in this review, 16 (59.2%) had app adoption as the final outcome, while 11 (40.7%) analyzed the app adoption intention as the final outcome. Those final outcomes may be related to the time of the studies. Most of the studies (7 studies or 63.6%) with app adoption intention as the final outcome were published in 2020, while three quarters (12 studies) having app adoption as the outcome were published in 2021.

**Table 1:** Studies included in the systematic review.

No	Study	Country	Study design	Sample size	Sampling design	Theory	Final outcome	Name of country COVID-19 Contact Tracing App
1	Altmann et al. (15)	France, German, Italy, United Kingdom, and United States	Cross-sectional	5,995	Stratified random sampling	n.a.	App adoption intention	TousAntiCovid (France), Corona Warn-App (German), Immuni (Italy), NHS COVID-19 app (United Kingdom), DCT apps
2	Hargittai et al. (16)	United States	Cross-sectional	1,254	Quota sampling method	n.a.	App adoption	DCT apps
3	Camacho-Rivera et al. (17)	United States	Cross-sectional	10,760	Probability sampling	n.a.	App adoption intention	DCT apps
4	Sharma et al. (18)	Fiji	Cross-sectional	714	Purposive sampling	PFT, PMT, PC, HCD, TPB	App adoption intention	CareFiji
5	Blom et al. (19)	German	Cross-sectional	3,276	Systematic sampling	n.a.	App adoption	Corona Warn-App
6	O’Callaghan et al. (20)	Republic Ireland	Cross-sectional	8,088	Systematic sampling	n.a.	App adoption intention	COVID Tracker
7	Garrett et al. (21)	Australia	Repeated cross-sectional	429–1,169	Purposive sampling method	n.a.	App adoption intention	COVIDSafe app
8	Abuhammad et al. (22)	Jordan	Cross-sectional	1,654	A convenience sample method	n.a.	App adoption	Aman.jo
9	Wnuk et al. (23)	Poland	Cross-sectional	1,033-1,046	Purposive sampling method	n.a.	App adoption	Kwarantanna domowa” app & “STOP COVID - ProteGO Safe” app
10	Jonker et al. (24)	Netherland	Discrete choice experiment	900	Purposive sampling method	n.a.	App adoption intention	Netherland COVID-19 contact tracing apps
11	Horvath et al. (25)	United Kingdom	Conjoint experiment	809–1,504	Purposive sampling method	n.a.	App adoption intention	NHS COVID-19 app
12	Dowthwaite et al. (26)	United Kingdom	Cross-sectional	1,001	Quota sampling method	TAM2	App adoption	NHS COVID-19 app
13	Duan and Deng (27)	Australia	Cross-sectional	307	a convenience sample	UTAUT, PCT	App adoption	COVIDSafe app
14	Guazzini et al. (28)	Italy	Cross-sectional	501	Stratified random sampling	TRA, TAM, UTAUT	App adoption	Immuni
15	Li et al. (2)	United States	Experimental design	1,963	Quota sampling method	URT, TAM	App adoption intentions	DCT apps
16	Nguyen et al. (29)	United States	Cross-sectional	288	a multi-stage sampling design	TAM	App adoption	COVID Alert PA and Covid Watch Arizona
17	Nurgalieva et al. (30)	United States, United Kingdom, Republic Ireland	Cross-sectional	871	Quota sampling method	n.a.	App adoption intentions	COVID Tracker (Ireland), DCT apps (United States), NHS COVID-19 app (United Kingdom)
18	Oldeweme et al. (31)	German	Cross-sectional	1,003	Simple random sampling	URT, TAM	App adoption	Corona-Warn-App
19	Saw et al. (32)	Singapore	Cross-sectional	505	A convenience sampling	n.a.	App adoption	TraceTogether
20	Sharma et al. (33)	India (New Delhi)	Repeated cross-sectional	28,246	a multi-stage sampling design	n.a.	App adoption	The Aarogya Setu application (ASA)
21	Tomczyk et al. (34)	German	Cross-sectional	349	a convenience sample	HBM, PMT, TPB, UTAUT1, UTAUT2	App adoption intentions	Corona Warn-App
22	Walrave et al. (35)	Belgium	Cross-sectional	1,500	Stratified random sampling	UTAUT	App adoption intentions	Coronalert
23	Shoji et al. (36)	Japan	Cross-sectional	7,084	Stratified random sampling	n.a.	App adoption	COCOA-COVID 19 Contact app
24	Fox et al. (37)	Republic Ireland	Longitudinal studies	405	Stratified random sampling	TAM, PCT, SET	App adoption	COVID Tracker
25	Huang et al. (38)	Singapore	Cross-sectional	3,240	A convenience sample method	n.a.	App adoption	TraceTogether
26	Tauzani et al. (39)	France	Cross-sectional	1,003	A quota sampling method	n.a.	App adoption	TousAntiCovid
27	Panchal et al. (40)	United Kingdom	Cross-sectional	1.036	A convenience sample method	n.a.	App adoption	NHS COVID-19 app

### 3.3 Quality assessment

The subjective quality assessment of the studies was generally positive (Supplementary Table S2). We did not think any of them were particularly prone to bias or had significant applicability issues. However, for at least one area, we assessed 8 of the 27 studies as ‘high risk’, either because there was insufficient information to assess the category or because we could not be sure what influence the reported technique for that category would have on bias.

### 3.4 Facilitators and barriers of mHealth adoption for COVID-19 contact tracing apps

The results of each study are summarized in Table 2. We reported based on each factor identified within the facilitators-barriers to adopt the mHealth framework. Facilitators are factors that increase the likelihood of contact tracing apps adoption, while barriers are factors that decrease the likelihood of contact tracing apps adoption. Table 3 shows the respective factors for each of the seven categories elicited from the 27 included articles. Social demographics out to be the most frequent category, while perceived benefits and technologies readiness the least frequent category.

**Table 2:** Studies included in the systematic review of facilitators and barriers of mHealth adoption for COVID-19 contact tracing apps.

No	Study	Country	Country classification	Perceived risks to COVID-19	Data privacy/ security concern	Trust	Perceived benefits	Social norm	Technology readiness	Social demographics
1	Hargittai et al. (16)	United States	High income economies	Yes	n.a	Yes	n.a	n.a	Yes	Yes
2	Camacho-Rivera et al. (17)	United States	High income economies	Yes	n.a	n.a	n.a	n.a	n.a	Yes
3	Li et al. (2)	United States	High income economies	Yes	Yes	n.a	Yes	Yes	Yes	Yes
4	Nguyen et al. (29)	United States	High income economies	Yes	n.a	n.a	Yes	na	Yes	n.a.
5	Horvath et al. (25)	United Kingdom	High income economies	n.a	Yes	Yes	n.a	n.a	n.a	n.a
6	Dowthwaite et al. (26)	United Kingdom	High income economies	n.a.	Yes	Yes	Yes	Yes	Yes	n.a
7	Panchal et al. (39)	United Kingdom	High income economies	n.a	Yes	n.a	n.a	n.a	Yes	n.a
8	Blom et al. (19)	German	High income economies	n.a	n.a	n.a	n.a	n.a	Yes	Yes
9	Oldeweme et al. (31)	German	High income economies	Yes	Yes	Yes	Yes	Yes	n.a	Yes
10	Tomczyk et al. (34)	German	High income economies	n.a	Yes	n.a	Yes	Yes	Yes	n.a.
11	Guazzini et al. (28)	Italy	High income economies	Yes	Yes	Yes	Yes	Yes	n.a	n.a
12	Tauzani et al. (2021)	France	High income economies	Yes	n.a	Yes	Yes	n.a	n.a	Yes
13	O’Callaghan et al. (20)	Republic Ireland	High income economies	Yes	n.a	n.a	n.a	n.a	n.a	Yes
14	Fox, et al. (37)	Republic Ireland	High income economies	n.a	Yes	n.a	Yes	Yes	n.a	Yes
15	Walrave et al. (35)	Belgium	High income economies	Yes	Yes	n.a	Yes	Yes	Yes	Yes
16	Jonker et al. (24)	Netherland	High income economies	n.a	Yes	n.a	n.a	n.a	n.a	n.a
17	Altmann et al. (15)	France, German, Italy, United Kingdom, and United States	High income economies	n.a	n.a	Yes	n.a	n.a	n.a	Yes
18	Nurgalieva et al. (30)	United States, United Kingdom, Republic Ireland	High income economies	n.a	n.a	Yes	n.a	Yes	n.a	n.a.
19	Shoji et al. (36)	Japan	High income economies	Yes	n.a	Yes	n.a	Yes	n.a	Yes
20	Garrett et al. (21)	Australia	High income economies	n.a	Yes	Yes	n.a	n.a	n.a	n.a
21	Duan and Deng (27)	Australia	High income economies	n.a	n.a	n.a	Yes	Yes	n.a	n.a
22	Saw et al. (32)	Singapore	High income economies	n.a	n.a	Yes	n.a	Yes	n.a	Yes
23	Huang et al. (38)	Singapore	High income economies	n.a	n.a	n.a	n.a	n.a	n.a	Yes
24	Wnuk et al. (23)	Poland	High income economies	Yes	Yes	n.a	n.a	n.a	n.a	Yes
25	Sharma et al. (18)	India (New Delhi)	Lower middle-income economies	n.a	n.a	n.a	n.a	n.a	Yes	Yes
26	Abuhammad et al. (22)	Jordan	Lower middle-income economies	n.a	Yes	n.a	n.a	n.a	n.a	Yes
27	Sharma et al. (33)	Fiji	Upper middle-income economies	n.a	n.a	n.a	n.a	Yes	Yes	n.a

**Table 3:** Summary of the facilitators and barriers of contact tracing apps adoption intention and contact tracing app adoption.

Factor	Facilitator	Barrier	No association
Perceived risks to COVID-19 (#Article = 11)			
perceived risk to COVID-19	(2, 16, 17, 28, 29, 36, 40)		(31, 35)
COVID-related anxiety	(41)		
sense of personal threat and a loss of personal control	(23)		
Data privacy/security concerns (#Article = 13)			
reduction in privacy risks	(24, 31, 34)		
Privacy concerns		(2, 22, 23, 26, 28, 35, 37, 39)	(25)
Secondary data use risks		(2)	
Security concerns		(21)	
Trust (#Article = 11)			
trust in government	(15, 28, 30, 31, 36, 40)	(21)	(16)
Trust in medical system	(25, 26, 30)		(16)
Trust in other organizations	(30, 31, 40)		(16)
Perceived benefits (#Article = 10)			
reduction of performance risks	(31, 35)		
perceived utility	(2, 26, 28, 34, 40)		
perceived health benefits	(29, 35, 37)		
Benefit outweigh the risk	(27)		
Social norm (#Article = 12)			
lower social risks			(31)
perceived societal expectations	(2, 18, 27, 30, 34, 35)		
Protect the society	(26, 32, 36, 37)		
Protect families and important persons	(28)		
Technology readiness (#Article = 10)			
technology readiness and protection	(2, 18)		
internet skill	(16, 27)		
perceived ease of use	(27)		(29, 34, 39)
individual innovativeness	(35)		
Have no phones and difficulties in using the apps		(38)	
Socio-demographics (#Article = 16)			
Age	(15, 16, 32)	(2, 19, 23, 31, 33, 38)	(35–37)
Gender		(2, 23, 33, 41)	(15, 19, 31, 32, 35–37)
Income	(22)	(2, 33, 40)	
Education	(16)	(2, 33)	(19, 31, 32, 35, 36–37)
Ethnicities	(17)		(32)
Living areas	(22)		
Marital status			(32)

### 3.5 Perceived risks to COVID-19

We found 11 studies among 27 selected studies that include perceived risks as one of variables to explain COVID-19 app adoption. Among those 11 studies, 9 reported a significant association of perceived risks to app adoption. In high income economies contexts, Li et al. (2), Nguyen et al. (29), Camacho-Rivera et al. (17), and Hargittai et al. (16) reported that perceived risk to COVID-19 is associated with app adoption in the United States. In Italy, Guazzini et al. (28) reported that risk perception of COVID-19 infection was associated with apps uptake. In the Republic of Ireland, O’Callaghan et al. (41) found that COVID-related anxiety was linked to a desire to install the app. in France, Touzani et al. (40) found that feeling concerned and perceived risk about the pandemic situation is associated with adoption intention. In Poland, Wnuk et al. (23) found acceptance of the COVID-19 contact tracing app was highly correlated with a sense of personal threat and a loss of personal control. In Japan, Shoji et al. (36) found that concerns about health risks determined the mHealth uptake decision. In contrast, Walrave et al. (35) and Oldeweme et al. (31) reported that found no association between COVID-19 concern on apps adoption in German.

### 3.6 Data privacy/security concerns

Among 27 selected studies, 13 studies included data privacy or security concerns in their estimation, and 12 of them reported a significant association of data privacy and security concerns on COVID-19 contact tracing adoption. In high income economy contexts, Oldeweme et al. (31) reported that adoption is linked to a reduction in privacy risks (i.e., individuals are concerned about data security, such as possible data leaks or misuse by third parties) in Germany. In another study in German, Tomczyk et al. (34) also found a trade-off between mHealth user concerns and perceived security on adoption intentions. In the United States study, Li et al. (2) found that privacy concerns generally had a significant negative effect on adoption, particularly the decision to install the apps. Secondary data use risks were also associated with lower app installation. However, no significant association was discovered between data breach risk and the risk of COVID-19 positive users being re-identified after implementation. In a Belgium study, Walrave et al. (35) found that app-related privacy concern was negatively associated with behavioral intention. In an Italy study, Guazzini et al. (28) found attitudes toward contact tracing systems that capture question such as “I’m afraid of contact tracing systems” and “Contact tracing systems excessively violate privacy” was negatively associated with adoption. In Republic Ireland study, Fox et al. (37) concerns about privacy have a detrimental, albeit little, impact on willingness to trust the app. In the United Kingdom, Panchal et al. (39) reported that privacy concerns were a possible reason why users did not download it. In a United Kingdom study, Dowthwaite et al. (26) also reported that privacy concern was associated with adoption intentions. However, in an experimental study, Horvath et al. (25) found privacy issues did not have as much of an impact on the digital app selection as previously thought or indicated by a study in the United Kingdom. In a study in Australia, Garrett et al. (21) showed that low uptake was linked to concerns about data security (e.g., maintaining privacy). In Netherland, Jonker et al. (24) found a contact tracing app that is both secure and private and has the most realistic features that can achieve a high adoption rate. Wnuk et al. (23) found a potential threat to privacy and other civil rights is associated with less acceptance of contact tracing apps in Poland. In lower-middle income economy contexts, Abuhammad et al. (22) the privacy of the data was a major ethical concern for many of the participants in Jordan.

### 3.7 Trust

We documented 11 studies that examined the relationship of trust to adoption. All those studies were in high income economy. Among them, only one study reported a null significant association of trust to adoption. In a study in the United States, Hargittai et al. (16) reported that trust in the medical system, federal gov, local gov, and business leaders was not associated with United States adoption intention. Other studies show a significant association of trust to adoption. In contrast, Altmann et al. (15) and Guazzini et al. (28) found that trust in the government was associated with adoption in France, Germany, Italy, United Kingdom, and United States. Likewise, Nurgalieva et al. (30) in a study involving United States, United Kingdom, and Republic Ireland respondents, documented that trust in current government and trust in politicians in the country where respondents are living, trust in medical doctors and nurses, trust in religious organizations, trust the accuracy of news from political party and leaders, and trust in religious organizations were associated with adoption intention. Dowthwaite et al. (26) found that trust in the NHS COVID-19 app is associated with the adoption of the NHS COVID-19 contact tracing app. Trust in the NHS United Kingdom has won out over privacy concerns, and the NHS centralized app is favored over both the centralized government app and the decentralized system (25). In German and France, Oldeweme et al. (31) and Touzani et al. (40) reported trust in government and trust in political representatives was indirectly associated with apps uptake. Saw et al. (32) reported that confidence in the government was associated with apps uptake in Singapore. In Japan, Shoji et al. (36) found that trust in national were key determinants of mHealth uptake for those aged between 40 and 59 years. A belief that the government was not trustworthy is associated with low uptake in Australia (21).

### 3.8 Perceived benefits

Ten studies included perceived benefits variables in their study. All of them were in high income economy. All those studies reported a significant association of perceived benefits measures on adoption. In German, Oldeweme et al. (31) found that reduction of performance risks (i.e., individuals are concerned that the product performs as it was designed and advertised) is associated with adoption. Tomczyk et al. (34) also found that perceived utility and hedonic motivation were both highly significant and consistent indicators of adoption intentions, but they were negative predictors of current app usage frequency. In the United States, Nguyen et al. (29) found perceived usefulness and health information orientation were associated with adoption intention. Li et al. (2) found that more prosocial people are significantly more inclined to install and use contact-tracing apps than those less prosocial in the United States. Guazzini et al. (28) found that positive perception of ‘Immuni’ apps was associated with adoption in Italy. Higher perceived usefulness is associated with adoption intention in French (40). Dowthwaite et al. (26) found that positive perception of NHS apps was associated with adoption in the United Kingdom. The early acceptability of citizens is influenced by their beliefs of health benefits in the Republic of Ireland (37). In Belgium, Walrave et al. (35) reported that performance expectancy (i.e., improving individual knowledge about the danger of being infected by COVID-19, which is beneficial for monitoring his/her risk of infection and controlling COVID-19 spread) is linked to behavioral intention. In Australia, Duan and Deng (27) reported that adoption was linked to the perceived value of information disclosure (i.e., when an individual feels that the risk of using an app is lower than the benefits, value given worth the disclosure of information, and benefits can outweigh the risk).

### 3.9 Social norm

Twelve studies examine the relationship of the social norm to adoption. Among those studies, one study reported a null association. In high income country economy, Oldeweme et al. (31) reported there was no link between lower social risks (i.e., individuals may be concerned about losing status in their social group if they use or do not use the app) and adoption in Germany. In contrast, another study in German by Tomczyk et al. (34) found a very strong connection between injunctive social norms (i.e., referring to perceived societal expectations) and adoption intentions. Walrave et al. (35) reported that social influence was a substantial predictor of adoption in Belgium. Nurgalieva et al. (30) documented that respondents’ perception that their actions to limit coronavirus spread make a difference linked with apps adoption in the United States, United Kingdom, and Republic Ireland. Dowthwaite et al. (26) reported that to help the NHS, protect others, and reduce the spread of the virus are the main reason to adopt NHS COVID-19 apps. in Italy, Guazzini et al. (28) found that individuals who know important others who have downloaded and installed the Immuni app are more likely to download and utilize the app themselves. In the United States, Li et al. (2) reported that ‘prosocialness’ is associated with contact tracing app acceptance. The views of social influence determine citizens’ initial acceptance of mHealth app in the Republic of Ireland (37). In Singapore, Saw et al. (32) found that using hand sanitizers, avoiding public transportation, and preferring outdoor over indoor settings are all linked to app use. In Japan, Shoji et al. (36) reported that concern about social risk was the key determinant of mHealth uptake for a senior adult. However, attachment to the community was the main driver to uptake the app for the middle-age. In Australia, Duan and Deng (27) documented that social influence as measured by reasons of individual used apps (i.e., recommended by the influential people, recommended by the important people, and recommended by the trusted people) are associated with adoption. In upper middle income economy contexts, Sharma et al. (18) reported that social normative pressures perceived by individuals were associated with app uptake in Fiji. Accordingly, they also found that in cultures with a high level of collectivism, the link between privacy concerns and attitude is weaker.

### 3.10 Technology readiness

Ten studies examined technology readiness in their study. Among those studies, we found 6 studies that reported a significant association of technology readiness on adoption. Using Parasuraman and Colby, Li et al. (2) documented that technology readiness was associated with app adoption in the United States. Hargittai et al. (16) reported that internet skill is associated with COVID-19 app contact tracing in the United States. However, in another study in the United States, Nguyen et al. (29) reported that perceived ease of use was not associated with app adoption in the United States. In the United Kingdom, Panchal et al. (39) found that little understanding regarding the app’s functionality is associated with less intention to uptake. Walrave et al. (35) reported that individual innovativeness and facilitating conditions were associated with using the app in Belgium. Tomczyk et al. (34) also found that perceived barriers, experience, and ease of use were not significant to COVID-19 app adoption in German. Likewise, in German, Blom et al. (19) reported potential spreaders have a high level of access to install the app, as well as a high level of competence to do so, but a low willingness to correctly adopt the app. In Australia, Duan and Deng (27) found that effort expectancy was measured by individual perception of easy to use, easy to become skillful, interaction is clear and understandable, and not much effort involved was associated with adoption. In Singapore, Huang et al. (38) documented that the app was not adopted by older persons who did not have cellphones, and those who did may have had difficulty installing and utilizing the program. In upper middle income economy contexts, Sharma et al. (18) found apps adoption was associated with habit describes the prior experience and individuals’ belief in their ability to protect their information privacy.

### 3.11 Socio-demographics

Among 16 selected studies, 15 studies include socio-demographic variables. In the context of high-income economy, Li et al. (2) documented females, lower income, lower education, and an older individual had lower intentions to adopt the app than males, higher income, higher education, and young individual. The significant association of race mostly appeared on the intentions to keep the app installed rather than the intentions to install the app, with Hispanics having much greater intentions to install than other races (17). Lower financial deprivation was associated with lower uptake in France (40). Walrave et al. (35) reported no association of health condition, age, gender, and education on behavioral intention. Saw et al. (32) found age-associated with apps uptake. However, gender, citizenship, household type and size, education, ethnicity, marital status were not significantly associated with app uptake in Singapore. Hargittai et al. (16) found that age and education are associated with adoption in the United States. However, female, urban, and household income are not. Altmann et al. (15) found young aged 18–30 more likely to uptake the apps than age 17 or lower in their cross-country data analyzes. A null association was found on the relationship between gender and app uptake. Males are more likely to say they will not install the app, either probably or definitely (41). The oldest and youngest generations are the most likely to say they will probably or definitely install the app (41). However, another study in Republic Ireland showed that age, gender, education, and health status all showed no correlations (37). The app was less likely to be downloaded and used by older persons in Singapore (38). Accordingly, Shoji et al. (36) found that age, female, and marriage status were not related to uptake in Japan. In Poland, Wnuk et al. (23) found male and older people are less likely accept contact tracing apps. Oldeweme et al. (31) and Blom et al. (19) reported that older individuals had lower intentions to use the apps. No associations were shown for education and gender. In the context lower middle income economy, Sharma et al. (33) reported the absence of an ASA installation was linked to being over 40 years old, being female, having fewer years of schooling, and having a lower *per capita* income (≤5,000 Indian National Rupee) in New Delhi India. In the context of upper middle income economy, acceptance and use of tracking technologies among Jordanians were predicted by income and living area in Jordan (22).

## 4 Discussion

This systematic literature review aims to identify facilitators and barriers to adopting mHealth apps for COVID-19 contact tracing. Our literature search returned 27 published manuscripts, of which 18 of them (66.67%) were from high-income economies. Prior reviews identified the low adoption of mHealth in countries with low-resource environments (42, 43). Research on mHealth apps adoption in low and middle-income economies is required as we found that the socio-demographic determinants, which include being older, females, having lower education attainment, and having lower income, are barriers for adoption only in low-middle income countries. Most of those factors were not significantly associated with adoption in high-income economies (18). In a study in China, the effect of perceived vulnerability and severity on attitude is more positive among women and the older adult than among men and teenagers, according to a Chinese study. As a result, they are more inclined to use the app to maintain their health (44). A study in Vietnam further shows that individuals born between 1981–1996 are the most potential COVID-19 app users because they are responsible for caring for their family health (29). The rise of information and communication technologies (ICTs) in low and middle-income nations has been hailed as a key step toward resolving many of these countries’ problems of underdevelopment. However, one important issue that these countries face is the so-called “digital divide” (45–47). Thus, addressing the digital divide is critical to the adoption of the app.

The highest proportion of the studies found a positive association between individual perception of the positive consequences of contact tracing apps and app uptake. We identified two positive benefits of the contact tracing app. First, individual safety benefits are associated with the app’s ability to detect possible contaminated person interactions and get exposure notifications. Individual advantages include being notified of a safe location (29, 31, 34). Second, societal benefits, if a user tests positive for COVID-19, the app notifies their most recent contact, protecting family, friends, and the general public from infection (28, 29, 31). Trang et al. (48) found that emphasizing the societal benefits of the app led to a higher adoption willingness than adoption intention was higher when the app’s societal benefits were highlighted rather than the app’s benefits to users. Because more prosocial people are likely to care more about contributing to the “greater good,” marketing these apps to appeal to this aspect could encourage uptake of contact tracing apps. Using health belief models, Walrave et al. (49) found that both individual and social perceived benefits respondents find in using the COVID-19 app are the most important predictor for intention to use the app.

Previous research suggests that social influence is important in the early stages of an individual’s experience with new technology, but that its importance diminishes over time and eventually fades away once the technology is used regularly, as one’s own experience provides a more instrumental basis for an individual’s continued use of the technology (50). In this study, the role of the social norm on app uptake was identified. Sharma, et al. (18) reported that social normative pressures perceived by individuals were associated with adoption. They also discovered that in societies with a high level of collectivism, the link between privacy concerns and attitude is weaker. Tomczyk et al. (34) found a strong connection between injunctive social norms and adoption intentions. Duan and Deng (27) documented that social influence as measured by reasons of individual used apps (i.e., recommended by the influential people, recommended by the important people, and recommended by the trusted people) are associated with adoption. Shoji et al. (36) highlighted attachment to the community plays a pivotal role in uptake among young people, while concern about the social impact of COVID-19 was an important factor for uptake for senior citizen in Japan.

Because contact-tracing applications are a new technology intended to supplement the traditional manual contact-tracing process, people’s views toward new technologies may have a significant impact on their acceptance. Duan and Deng (27) found that effort expectancy is measured by individual perception of easy to use, easy to become skillful, interaction is clear and understandable, and not much effort involved was associated with adoption. Sharma et al. (18) found apps adoption was associated with habit describes the prior experience and individuals’ belief in their ability to protect their information privacy in Fiji. Hargittai et al. (16) reported that internet skill is associated with COVID-19 app contact tracing in the United States.

Our finding further shows that data privacy and security concerns were the main barriers to app uptake adoption. Individuals are afraid of contact tracing systems due to excessively violating privacy and the possibility of data leaks or misuse by third parties (31). Li et al. (2) highlight that despite all of the technical safeguards in place to protect users’ privacy, the nature of contact tracing apps implies that, regardless of the app’s design, some security concerns are unavoidable. They found that only a few contact tracking app studies explicitly highlighted the security risks to their users and that they mostly focused on a certain form of security risk that is less protected against in a particular app design. For example, the risk of infected individuals being re-identified, which decentralized apps are more vulnerable to, and how users prefer centralized apps to decentralized apps. However, when users were prompted with the potential of a data breach, which centralized apps are also more subject to, the data breach stimuli did not change users’ preferences for data storage.

A review by Nurgalieva et al. (20) emphasized that the development of mHealth applications should comply with national and international regulations to ensure the privacy and security of users’ data. It also provided data security and privacy evaluation tools to help users maintain their privacy and security. Technical methods include studying code or performing traffic analysis to uncover potential data leaks, as well as non-technical methods, including evaluating an app based on user ratings.

Despite COVID-19 being a highly infectious disease, perception of risk to the disease varies greatly (51). This review found that perceived risks to COVID-19, trust, perceived benefit, social norm, and technology readiness are among the facilitators to apps adoption. Past pandemics have shown us that public perceptions of disease dangers have a considerable impact on the success of containing the spread of a highly contagious disease (52, 53). This review also found a significant association of perceived risks to COVID-19 on adoption. Individuals who perceive the risk of COVID-19 infection are likely to uptake the apps. However, some research brings up the issue of conspiracy theories concerning the seriousness of COVID-19, which operate as roadblocks to implementing disease-control measures like contact tracing app adoption (2).

Trust and confidence in the government and health system are strongly associated with adoption. This finding support prior research in the Netherlands, which found that trust was positively correlated to the intention to use mHealth applications (54). As security and privacy are an important concern for people, trust and confidence can be seen as a mechanism to reduce uncertainty and complexity of the issue around contact tracing apps’ security and privacy (31). For example, trust is highly important when individuals lack the knowledge to decide to use the apps, and trust reduces psychological reactance against public policies regarding the pandemic (31). During the SARS outbreak in Hong Kong and the H1N1 influenza pandemic in the Netherlands, previous research found that trust was critical in the adoption of preventative measures and adherence to government regulations (55, 56). According to a study done in the United Kingdom, people who have a higher level of trust in the responsible authorities are more likely to accept their advice (26).

Contact tracing applications were made available on different dates for each country, which may have an impact on adoption rates. For example, Italy started the first version of Italian Digital Contact Tracing in May 2020 (57), while Australia released COVIDSafe on April 26th, 2020 (58). The adoption of the contact apps may also affected by the vaccine availability. The adoption of the digital vaccine certificate has risen after the COVID-19 vaccine became available in 2021. The digital vaccine certificate was mostly included in the contact tracing apps (59), which may increase the adoption of the apps as people use them for “immunity passports” to move between countries or areas’ borders (60).

Taken together, the findings of this review highlight several implications regarding the adoption of digital technologies of the existing COVID-19 mHealth contact tracing and the future development of digital health pandemics. First, one of the important roles of mHealth technology in combating the COVID-19 pandemic is contact tracing. As COVID-19 contact-tracing apps warn people about the infection by identifying high-risk areas to refrain from commuting in those areas and give instructions to people who have recently been in contact with an infected person to pay attention to the symptoms, the success of the apps depend on public choice. The following are some recommendations arising from our review to inform decisions on enhancing the use of an mHealth app for COVID-19 contact tracing. One of the reasons people are willing to use an mHealth app for COVID-19 contact tracing demonstrates a keenness to help themselves, their families and friends, society, and the government to avoid the virus. However, they might change their decision to use the apps due to the concern of data security and privacy and the efficacy of the app. It is thus crucial to regularly update reliable information in mHealth. Second, another reason is the mistrust in the government. Policymakers should thus demonstrate trustworthiness and appoint a highly reputable and transparent public health authority to organize the mHealth app for COVID-19 contact tracing. Media could be used to provide citizens with credible sources of verified information. Finally, the digital divide, especially among older people, might affect the use of technology during COVID-19, including the adoption of mHealth. Greater public involvement in the development and implementation of policy and technologies is thus required from the outset and on an ongoing basis to ascertain the adoption of the app in whole population.

## 5 Conclusion

The findings suggest addressing issues of data privacy/security while fostering trust, perceived benefits, social norms, and technology readiness might be promising strategies to foster adoption intentions and app use in the general population. In low-middle-income countries, addressing digital divide is critical to the app’s adoption. The scope of this study was limited to facilitators and barriers within the adoption of mHealth framework and comprised seven key factors. Future studies may use other mHealth frameworks to enrich understanding of facilitators and barriers of mHealth adoption for COVID-19 contact tracing. Moreover, since most of the selected studies in the review employed a cross-sectional design, the findings should be seen as an association rather than causation. More studies based on longitudinal design and clinical trials are needed to address causality issues in the future.

## Data availability statement

The original contributions presented in the study are included in the article/Supplementary material, further inquiries can be directed to the corresponding author.

## Author contributions

SS and AM: conception or design of the work, data collection, data analysis, interpretation, drafting the article, and critical revision of the article. All authors contributed to the article and approved the submitted version.
