# Supplementary material for: Facilitators and barriers to the adoption of mHealth apps for COVID-19 contact tracing: a systematic review of the literature
Source: Front Public Health. 2023 Dec 7;11:1222600. doi: 10.3389/fpubh.2023.1222600 (PMC10740170; doi:10.3389/fpubh.2023.1222600)
Supplement: Supplementary file 3 [file Table_3.DOCX]

**S3. Table 2.** Risk of bias summary for studies included in the systematic review

| No | Study | Country | Recruitment bias | Missing outcome data | Outcome measurement | Selective reporting bias | Overall bias |
| --- | --- | --- | --- | --- | --- | --- | --- |
| 1 | Altman, S., et al., 2020 | France, German, Italy, UK, and US | Low Risk | Low Risk | Low Risk | Low Risk | Low Risk |
| 2 | Hargittai, E. et al., 2020 | US | Low Risk | Low Risk | Low Risk | Low Risk | Low Risk |
| 3 | Camacho-Rivera, M. et al., 2020 | US | Low Risk | Low Risk | Low Risk | Low Risk | Low Risk |
| 4 | Sharma, S. et al., 2020 | Fiji | Low Risk | Low Risk | Low Risk | Low Risk | Low Risk |
| 5 | Blom, A.G. et. al., 2020 | German | Low Risk | Low Risk | Low Risk | Low Risk | Low Risk |
| 6 | O’Callaghan, M.E., et al. 2020 | Republic Ireland | Low Risk | Low Risk | Low Risk | Low Risk | Low Risk |
| 7 | Garret, P.M. et al., 2020 | Australia | Low Risk | Low Risk | Low Risk | Low Risk | Low Risk |
| 8 | Abuhammad, S., et al. 2020 | Jordan | High Risk | Low Risk | Low Risk | Low Risk | Low Risk |
| 9 | Wnuk, A. et al. 2020 | Poland | Low Risk | Low Risk | Low Risk | Low Risk | Low Risk |
| 10 | Jonker, M. et al, 2020 | Netherland | Low Risk | Low Risk | Low Risk | Low Risk | Low Risk |
| 11 | Horvath, L. et al. 2020 | UK | Low Risk | Low Risk | Low Risk | Low Risk | Low Risk |
| 12 | Dowthwaite, L. et al., 2021 | UK | Low Risk | Low Risk | Low Risk | Low Risk | Low Risk |
| 13 | Duan, S. X., & Deng, H., 2021 | Australia | High Risk | Low Risk | Low Risk | Low Risk | Low Risk |
| 14 | Guazzini, A., 2021 | Italy | High Risk | Low Risk | Low Risk | Low Risk | Low Risk |
| 15 | Li, T. et al., 2021 | US | Low Risk | Low Risk | Low Risk | Low Risk | Low Risk |
| 16 | Nguyen, T. et al., 2021 | US | High Risk | Low Risk | Low Risk | Low Risk | Low Risk |
| 17 | Nurgalieva, L., et al., 2021 | US, UK, Republic Ireland | High Risk | High Risk | Low Risk | Low Risk | Middle Risk |
| 18 | Oldeweme, A., et al., 2021 | German | Low Risk | Low Risk | Low Risk | Low Risk | Low Risk |
| 19 | Saw, Y.E., et al., 2021 | Singapore | High Risk | Low Risk | Low Risk | Low Risk | Low Risk |
| 20 | Sharma, N., 2021 | India (New Delhi) | Low Risk | Low Risk | Low Risk | Low Risk | Low Risk |
| 21 | Tomczyk, S., 2021 | German | High Risk | Low Risk | Low Risk | Low Risk | Low Risk |
| 22 | Walrave, M. et al., 2021 | Belgium | Low Risk | Low Risk | Low Risk | Low Risk | Low Risk |
| 23 | Shoji, M. et al., 2021 | Japan | Low Risk | Low Risk | Low Risk | Low Risk | Low Risk |
| 24 | Fox, G. et al., 2021 | Republic Ireland | High Risk | Low Risk | Low Risk | Low Risk | Low Risk |
| 25 | Huang, Z., et al. 2021 | Singapore | Low Risk | Low Risk | Low Risk | Low Risk | Low Risk |
| 26 | Tauzani, R. et al., 2021 | France | Low Risk | Low Risk | Low Risk | Low Risk | Low Risk |
| 27 | Panchal, M. et al. 2021 | UK | Low Risk | Low Risk | Low Risk | Low Risk | Low Risk |
